# Maladie de Bowen pigmentée génitale

**DOI:** 10.11604/pamj.2014.19.192.2629

**Published:** 2014-10-23

**Authors:** Mariem Bounouar, Fatimazahra Mernissi

**Affiliations:** 1 Service de Dermatologie, CHU Hassan II, Université Sidi Mohammed Ben abdellah, Fès, Maroc

**Keywords:** Maladie de Bowen, tumeurs, koïlocytes, Bowen disease, tumors, koilocytes

## Image en medicine

Il s'agit d'un patient de 59 ans, qui présentait de multiples lésions génitales évoluant depuis 2 ans. L'examen a objectivé 2 tumeurs verruqueuses superficielles, à bordure pigmentée et à surface végétante, siégeant respectivement au niveau sus-pubien et sur le pli inguinal droit; une macule pigmentée hétérogène au niveau hypogastrique, et de multiples plaques noirâtres homogènes à surface verruqueuse grasse au niveau pubien. L’étude histologique a trouvé une tumeur confinée à l’épiderme avec papillomatose, des cellules atypiques, des mitoses et quelques koïlocytes avec une membrane basale intacte. Nous avons retenu le diagnostic de maladie de Bowen (MB) pigmentée génitale, et le patient a bénéficié d'une exérèse chirurgicale des lésions volumineuses, et d'une cryothérapie pour les lésions de petite taille avec bonne évolution. La MB est un carcinome spinocellulaire in situ qui se présente typiquement sous forme de plaque érythémateuse bien limitée. La forme pigmentée est rare et la localisation génitale n'est pas fréquente. L'association de plusieurs formes cliniques chez notre patient suggère un spectre évolutif des lésions cutanées. L'infection à HPV, fortement suspectée dans ce cas vu la présence de koïlocytes, est parmi les facteurs étiologiques incriminés dans la survenue de MB dans les zones non exposées au soleil ainsi que les zones souvent infectées par l'HPV notamment la région génitale comme c'est le cas de notre malade.

**Figure 1:**
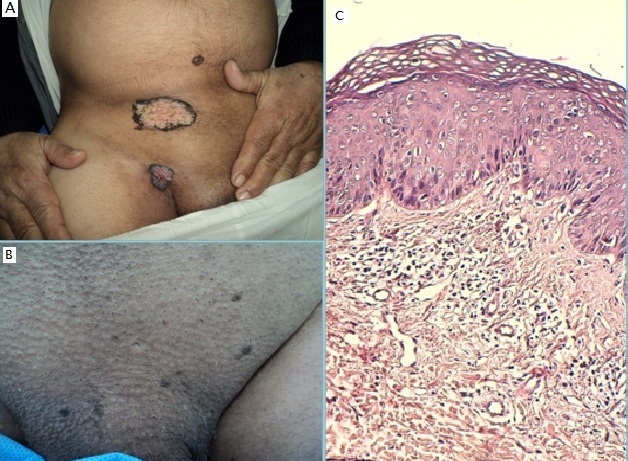
A) deux tumeurs verruqueuses à bordure pigmentée et macule pigmentée hétérogène; B) multiples plaques noirâtres verruqueuses pubiennes; C) Aspect histologique

